# Impact of 5-aminolevulinic acid with iron supplementation on exercise efficiency and home-based walking training achievement in older women

**DOI:** 10.1152/japplphysiol.00582.2015

**Published:** 2015-10-29

**Authors:** Shizue Masuki, Atsumi Morita, Yoshi-ichiro Kamijo, Shigeki Ikegawa, Yufuko Kataoka, Yu Ogawa, Eri Sumiyoshi, Kiwamu Takahashi, Tohru Tanaka, Motowo Nakajima, Hiroshi Nose

**Affiliations:** ^1^Department of Sports Medical Sciences, Shinshu University Graduate School of Medicine, Matsumoto, Japan;; ^2^Institute for Biomedical Sciences, Shinshu University, Matsumoto, Japan; and; ^3^Department of R&D, SBI Pharmaceuticals Co., Ltd., Tokyo, Japan

**Keywords:** 5-aminolevulinic acid, exercise efficiency, respiratory response, home-based walking training achievement

## Abstract

A reduction in exercise efficiency with aging limits daily living activities. We examined whether 5-aminolevulinic acid (ALA) with sodium ferrous citrate (SFC) increased exercise efficiency and voluntary achievement of interval walking training (IWT) in older women. Ten women [65 ± 3(SD) yr] who had performed IWT for >12 mo and were currently performing IWT participated in this study. The study was conducted in a placebo-controlled, double-blind crossover design. All subjects underwent two trials for 7 days each in which they performed IWT with ALA+SFC (100 and 115 mg/day, respectively) or placebo supplement intake (CNT), intermittently with a 2-wk washout period. Before and after each trial, subjects underwent a graded cycling test at 27.0°C atmospheric temperature and 50% relative humidity, and oxygen consumption rate, carbon dioxide production rate, and lactate concentration in plasma were measured. Furthermore, for the first 6 days of each trial, exercise intensity for IWT was measured by accelerometry. We found that, in the ALA+SFC trial, oxygen consumption rate and carbon dioxide production rate during graded cycling decreased by 12% (*P* < 0.001) and 11% (*P* = 0.001) at every workload, respectively, accompanied by a 16% reduction in lactate concentration in plasma (*P* < 0.001), although all remained unchanged in the CNT trial (*P* > 0.2). All of the reductions were significantly greater in the ALA+SFC than the CNT trial (*P* < 0.05). Furthermore, the training days, impulse, and time at fast walking were 42% (*P* = 0.028), 102% (*P* = 0.027), and 69% (*P* = 0.039) higher during the ALA+SFC than the CNT intake period, respectively. Thus ALA+SFC supplementation augmented exercise efficiency and thereby improved IWT achievement in older women.

aerobic capacity decreases by ∼10% every 10 yr after 30 yr of age with the progression of aging (2). A reduction in O_2_ utilization efficiency in the muscles has been suggested to be one of the mechanisms underlying this decrease in aerobic capacity (14, 48). Experimentally, the mitochondrial electron transport chain (ETC) function, especially complex IV (cytochrome *c* oxidase) activity, was reported to decline with aging in human (32, 45, 50) and animal muscles (13, 42, 49). The dysfunction of mitochondria has been suggested not only to decrease exercise efficiency (5, 10, 47), but also to enhance the generation of reactive oxygen species to injure the tissues (13, 42, 48, 49), which may evoke chronic inflammatory responses in the body and thereby cause lifestyle-related diseases (18). Thus the dysfunction of mitochondrial function may be one of the key mechanisms in limiting daily physical activity and evoking lifestyle-related diseases in middle-aged and older people (18, 19, 40).

To prevent this, aerobic exercise training above a given intensity has been recommended (1); however, during exercise, the ATP demand increases drastically in active muscles in the face of the decline in mitochondrial functions with aging (32, 45, 48, 50). Consequently, there is a huge imbalance between ATP production and demand (23), which may accelerate lactic acid production in muscles and thus the dissociation of hydrogen ions, evoking panting as compensatory hyperventilation for metabolic acidosis and sometimes causing pain through the muscle receptors (22). These responses may diminish the enjoyment of, and intrinsic motivation for, physical activity and may prevent middle-aged and older people from performing exercise training. Accordingly, we surmised that, if some nutritional supplements are proved to improve mitochondrial functions, they will help those who feel difficulty in performing exercise training to lower the psychological barrier.

One of the candidates for these supplements is 5-aminolevulinic acid (ALA). ALA is an amino acid that can be found in many foods and is the sole initial material of heme biosynthesis in vivo (15, 37, 39). It has been confirmed that ALA is incorporated into cytochromes *a*, *b*, and *c* in the mitochondrial ETC in vitro (37, 39). Indeed, it has been reported that the oral ingestion of ALA by mice increased complex IV activity and raised the ATP production rate in the liver (38). However, no studies have evaluated how ALA affects the age-related reduction in exercise efficiency and the voluntary achievement of exercise training.

Based on these facts, we hypothesized that, if the oral ingestion of ALA accelerated the O_2_ utilization efficiency in muscle mitochondria, it would increase the exercise efficiency and thereby improve the training achievement in older human subjects. To examine this hypothesis, we conducted a randomized, placebo-controlled, double-blind crossover study in older women.

## METHODS

### Subjects

This study was approved by the Review Board on Human Experiments, Shinshu University School of Medicine, and conformed to the standards set by the Declaration of Helsinki. To minimize the effects of subject selection and the levels of training readiness, the subjects were recruited from the 547 participants in the “Jukunen Taiikudaigaku Project,” which is a health promotion program for middle-aged and older people in Matsumoto City, Japan. The participants had performed the interval walking training (IWT) program for more than 12 mo before this study; therefore, they were familiar with the exercise testing procedures used in the present study. Moreover, their exercise efficiency had likely reached the steady state and would thus enable us to detect only the effects of ALA intake on their exercise efficiency without accounting for the acute effects of exercise training.

After the experimental protocol was fully explained, 10 healthy older female volunteers (60-69 yr old) gave written, informed consent before participating in this study. Each subject gave a complete medical history and underwent examinations of physical characteristics, resting blood pressure (BP), and peak aerobic capacity (V̇o_2 peak_). All subjects were nonsmokers and had no overt history of hepatic, thyroid, renal, metabolic, cardiovascular, or pulmonary disease, or orthopedic limitations in the exercise tests. Moreover, no subjects showed symptoms of iron deficiency anemia, as confirmed in the health examination before participating in the study. In the examination, the hemoglobin concentration ([Hb]), mean corpuscular volume, and mean corpuscular [Hb] were 14.1 ± 0.7 g/dl (mean ± SD), 90.5 ± 2.2 fl, and 33.8 ± 0.7 g/dl, respectively. Their physical characteristics were similar to those observed in our laboratory's previous study (34) and are summarized in Table 1.

**Table 1.** Physical characteristics of subjects

	CNT		ALA+SFC	
	Before	After	Before	After
Age, yr	65 ± 3	NA	NA	NA
Height, cm	153 ± 3	NA	NA	NA
Body weight, kg	52.6 ± 2.2	52.6 ± 2.1	52.8 ± 2.1	52.8 ± 2.1
BMI, kg/m^2^	22.5 ± 1.0	22.5 ± 1.0	22.6 ± 1.0	22.6 ± 1.0
HR_rest_, beats/min	64 ± 2	64 ± 2	62 ± 2	62 ± 2
SBP_rest_, mmHg	143 ± 5	135 ± 7	141 ± 6	137 ± 5
DBP_rest_, mmHg	82 ± 3	79 ± 4	82 ± 4	79 ± 3
V̇o_2peak_, ml·kg^−1^·min^−1^	25.0 ± 1.2	24.7 ± 1.1	25.4 ± 1.6	24.9 ± 1.5
HR_peak_, beats/min	150 ± 2	150 ± 2	148 ± 4	149 ± 2
WL_peak_, W	96 ± 5	96 ± 6	93 ± 4	98 ± 3
Time_exhaustion_, min	16.7 ± 0.6	16.4 ± 0.7	16.0 ± 0.7	16.8 ± 0.5*

Values are means ± SD for age and height, and means ± SE for the other variables; *n* = 10 subjects.

CNT, placebo intake condition; ALA+SFC, 5-aminolevulinic acid + sodium ferrous citrate intake condition; NA, not applicable; BMI, body mass index; HR_rest_, resting heart rate; SBP_rest_ and DBP_rest_, resting systolic and diastolic blood pressure, respectively; V̇o_2peak_, peak oxygen consumption rate during the graded cycling test; HR_peak_, peak heart rate at V̇o_2peak_; WL_peak_, peak workload at V̇o_2peak_; Time_exhaustion_, exercise time to exhaustion.

* Compared with before supplement intake, *P* < 0.05.

### Protocol

**Fig. 1.**
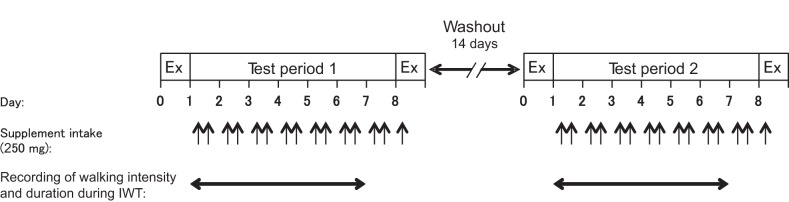
Experimental protocol. Ex, graded cycling exercise test; IWT, interval walking training. The study was conducted using a randomized, placebo-controlled, double-blind crossover design. In each supplement intake, subjects ingested 250 mg of either 5-aminolevulinic acid (ALA) + sodium ferrous citrate (SFC) or placebo supplement (250 mg × 2 = 500 mg/day). See Table 2 for details of the supplement compositions.

As shown in Fig. 1, this study was carried out in a randomized, placebo-controlled, double-blind crossover design. All subjects participated in two trials for 9 days each: 7 days of supplement intake, and 2 days of graded cycling tests, with a 14-day washout period between the trials. Subjects ingested either ALA [ALA + sodium ferrous citrate (SFC) trial, see below for details of SFC] or placebo supplement (CNT trial) two times per day for 7 days (*days 1–7*). Before and after the supplement intake period (*days 0* and *8*), subjects underwent a graded cycling test, during which the cardiorespiratory responses and lactate concentration in plasma ([Lac^−^]_p_) were measured. During the supplement intake period (*days 1–6*), except for the day before the graded cycling test (*day 7*), the training days, intensity, and time were recorded with a triaxial accelerometer (JD Mate; Kissei Comtec, Matsumoto, Japan) (30, 34, 52). The reason for no measurement on the 7th day was that we instructed subjects not to perform IWT on that day to avoid any effects of IWT on the results of the graded cycling test scheduled the next day.

### Supplements

The composition of supplements (SBI ALApromo, Tokyo, Japan) is shown in Table 2. Subjects ingested the supplements more than 1 h before breakfast and dinner for 7 days. The dose of ALA phosphate in the present study (100 mg/day) was higher than the doses used in previous studies (5–50 mg/day) that have examined the effects and safety of ALA supplementation in humans (21, 43, 51); however, those studies were performed on relatively sedentary subjects, whereas in the present study we examined the effects of ALA supplementation on exercise efficiency and training achievement when oxygen consumption rate (V̇o_2_) was increased. Therefore, we thought that a higher dose would be needed to improve mitochondrial function, if it occurred. Regarding dose safety, there is no legal dose limit in Japan because ALA phosphate has been approved as a food ingredient by the Pharmaceutical and Food Safety Bureau, the Ministry of Health, Labour and Welfare. Additionally, because the present study had a placebo-controlled crossover design, we could not test several doses of ALA+SFC.

**Table 2.** Composition of supplements

	Placebo Supplement (250.00 mg/dose)	ALA+SFC Supplement (250.00 mg/dose)
ALA phosphate, mg	0.00	50.00
SFC, mg	0.00	57.36
Pregelatinized starch, mg	247.50	140.14
Silicon dioxide mixture, mg	2.50	2.50

SFC, as a source of the iron ion, was contained in the supplements to enhance the final step of heme biosynthesis by ABCB6 transporter and ferrochelatase in mitochondria and to prevent the accumulation of heme biosynthesis intermediates, such as protoporphyrin IX, which might cause photodamage to the skin during outdoor exercise (39). Although SFC is used for anemia treatment in Japan, the dose used in the present study (12 mg iron/day) was much less than that used in regular treatment (100–200 mg iron/day), such that no significant effects on red blood cell production were expected during the 7-day intake period in healthy volunteers.

### Dietary Intake

Subjects in both conditions were instructed to maintain their dietary habits, except for the supplements, during the study period. Furthermore, they were instructed to report the food that they consumed during the 7-day supplement intake period in both trials by answering a questionnaire that was prepared by a dietician (FFQg version 3.5; Kenpakusya, Tokyo, Japan). The results are shown in Table 3. We confirmed that there were no significant differences in the values between the CNT and ALA+SFC trials (*P* > 0.2). Moreover, the values generally met the recommended dietary allowance (RDA) for Japanese active older women (29), and the amount of ALA contained in the diet, as calculated according to the references (15), was very low compared with that contained in the ALA supplement (Table 2).

**Table 3.** Total energy, protein, fat, carbohydrate, ALA, and iron intake per day during the supplement intake period

	CNT	ALA+SFC
Energy, kcal	1,629 ± 71	1,530 ± 75
Protein, g	59.7 ± 2.9	58.0 ± 3.7
Fat, g	48.7 ± 3.6	45.3 ± 2.6
Carbohydrate, g	232 ± 10	218 ± 11
ALA, μg	52.9 ± 4.3	53.3 ± 5.0
Iron, mg	6.5 ± 0.4	6.3 ± 0.5

Values are means ± SE; *n* = 10 subjects.

On the day before the graded cycling test, which occurred four times [(before and after the ALA or placebo intake periods)/subject × 10 subjects = 40 times, food was controlled over the course of the day (i.e., standardized breakfast, lunch, and dinner)]: the total calories was 1,816 ± 16 kcal, the total protein was 66.6 ± 0.3 g, the total fat was 33.3 ± 0.1 g, and the total carbohydrate was 307 ± 4 g, with no significant differences observed before and after the supplement intake periods or between the CNT and ALA+SFC trials (*P* > 0.4). Subjects were asked to eat the standardized breakfast and lunch at normal time and to finish the standardized dinner by 2100. Additionally, subjects were asked to refrain from ingesting alcohol and caffeine during the day.

### Graded Cycling Test

On the day of testing, subjects reported to the laboratory at 0900; they were normally hydrated but had not eaten any food for more than 12 h before the experiment. To ensure that they were well hydrated, they were instructed to drink 500 ml of tap water 2 h before the visit. Furthermore, they were instructed to ingest a supplement 2 h before the visit on *day 8* only. After emptying their bladders, they were weighed in the nude, put on light clothes and shoes, and entered an artificial climate chamber adjusted to 27.0 ± 0.1°C (mean ± range) atmospheric temperature and 50 ± 1% relative humidity. An 18-gauge Teflon catheter was then placed in the left antecubital vein for blood sampling. The catheter was connected to a 20-ml syringe that contained heparinized saline solution through an extension tube 2.0 ml in volume with a three-way stopcock.

**Fig. 2.**
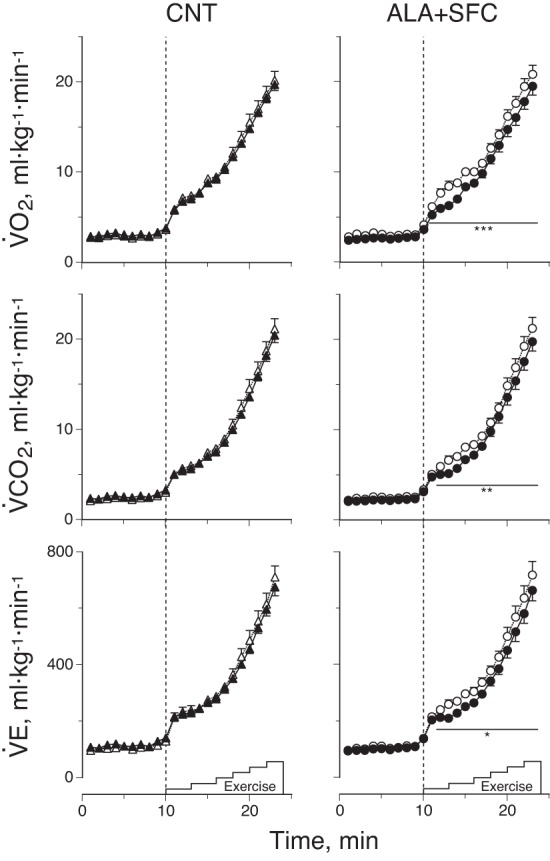
Oxygen consumption rate (V̇o_2_), carbon dioxide production rate (V̇co_2_), and ventilation volume (V̇e) responses during graded cycling exercise under placebo (CNT; *left*) and ALA+SFC supplement intake (*right*) conditions. The average value per minute is presented from rest to the highest workload of 75 W at which all subjects could maintain the rhythm. Open symbols, before supplement intake; solid symbols, after supplement intake. Values are means ± SE of 10 subjects. *P < 0.05, **P < 0.01, and ***P < 0.001 vs. before supplement intake.

Subjects rested quietly in a semirecumbent position in the contoured chair of the cycle ergometer for 60 min, while all of the measurement devices were applied. After resting baseline measurement was taken for 10 min, subjects performed the graded cycling exercise at 60 revolutions/min. After exercising at 0 W and 15 W for 3 min each, the intensity was increased by 15 W every 2 min until they could not maintain the rhythm due to exhaustion. We measured the respiratory gas fraction and ventilation volume (V̇e) every 15 s (Aeromonitor AE260; Minato, Tokyo, Japan) to determine the V̇o_2_ and carbon dioxide production rate (V̇co_2_) (Fig. 2, Table 4). In addition, we measured the heart rate (HR) using an electrocardiogram trace (Life Scope 8; Nihon Kohden, Tokyo) and systolic and diastolic BP from the right upper arm at the heart level by inflation of the cuff with sonometric pickup of Korotkoff's sound (STBP-780; Colin, Komaki, Japan) every minute.

**Table 4.** Changes in V̇o_2_, V̇co_2_, V̇e, and [Lac^−^]_p_ during the graded cycling test after supplement intake

	CNT		ALA+SFC		
	Change	*P* value*	Change	*P* value*	CNT vs. ALA+SFC *P* value
V̇o_2_, ml·kg^−1^·min^−1^	−0.17 ± 0.24	NS	−1.01 ± 0.17	<0.001	0.023
V̇co_2_, ml·kg^−1^·min^−1^	−0.19 ± 0.16	NS	−0.79 ± 0.17	0.001	0.036
V̇e, ml·kg^−1^·min^−1^	−4.8 ± 6.8	NS	−26.2 ± 9.5	0.022	NS
[Lac^−^]_p_, mmol/l	−0.04 ± 0.07	NS	−0.23 ± 0.04	<0.001	0.028

Values are means ± SE; *n* = 10 subjects. Average values during the graded cycling test are presented.

V̇o_2_, oxygen consumption rate; V̇co_2_, carbon dioxide production rate; V̇e, ventilation volume; [Lac^−^]_p_, lactate concentration in plasma.

* Before vs. after supplement intake.

#### V̇o_2 peak_ and gas exchange threshold.

The criteria used to determine V̇o_2 peak_ were a respiratory exchange ratio of >1.1, V̇o_2_ leveling off, despite increasing exercise intensity, and HR reaching the age-predicted maximal value. V̇o_2 peak_ was determined by averaging the three largest consecutive values at the end of exercise. We also determined the gas exchange threshold during graded cycling according to the standard method (3) from the V̇o_2_ vs. V̇co_2_ relationship. Peak HR was adopted at V̇o_2 peak_. Because the highest workload at which all subjects could maintain the rhythm for >1 min in the four graded cycling tests was 75 W, data were presented from rest to 75 W.

#### [Lac^−^]_p_.

**Fig. 3.**
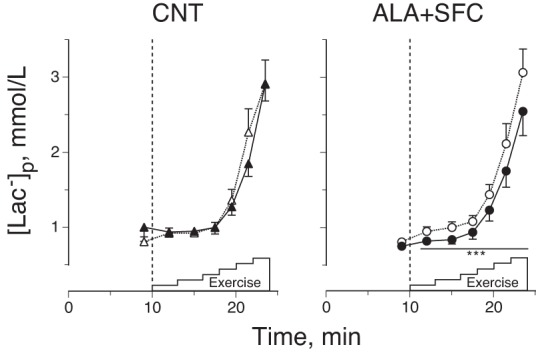
Plasma lactate concentration ([Lac^−^]_p_) responses during graded cycling exercise under CNT (*left*) and ALA+SFC (*right*) conditions. Open symbols, before supplement intake; solid symbols, after supplement intake. Values are means ± SE of 10 subjects. ***P < 0.001 vs. before supplement intake.

Blood samples were taken at rest and at the last minute of each intensity to determine [Lac^−^]_p_. An aliquot of the blood sample was transferred to a heparin-treated tube and centrifuged for 3 min at 8,000 rpm, and the separated plasma was stored at −85°C until the assays were performed. The plasma was used to determine [Lac^−^]_p_ (YSI 2300 Stat Plus; Yellow Springs, OH) (Fig. 3). Additionally, an aliquot of the blood sample at rest was transferred to a heparin-treated tube that was also used to determine [Hb] with the sodium lauryl sulfate hemoglobin method.

#### Exercise efficiency.

**Fig. 4.**
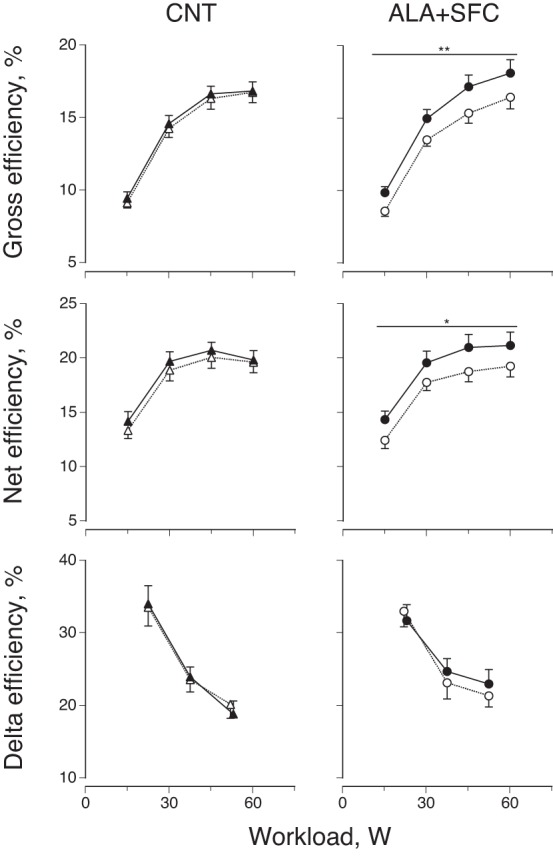
Gross, net, and delta efficiencies during graded cycling exercise under CNT (*left*) and ALA+SFC (*right*) conditions. Open symbols, before supplement intake; solid symbols, after supplement intake. Values are means ± SE of 10 subjects. *P < 0.05 and **P < 0.01 vs. before supplement intake.

V̇o_2_ and respiratory exchange ratio values during the last minute of each workload were averaged and used to calculate energy expenditure at a given workload. Under these conditions, efficiency was calculated based on the steady-state assumption that energy requirements are met by V̇o_2_ (6). In the present study, this was confirmed by the following observations: *1*) V̇o_2_ during the last minute of each workload was relatively constant; and *2*) when the values were plotted against workload, there was a linear relationship between them in each subject of each condition (all, *R*^2^ > 0.92, *P* < 0.009). Efficiency was then determined (Fig. 4) using the method reported by Gaesser and Brooks (16) with the following equations: gross efficiency = work accomplished/energy expended; net efficiency = work accomplished/energy expended above that at rest; delta efficiency = change in work accomplished/change in energy expended during graded exercise.

#### V̇o_2_ kinetics.

V̇o_2_ measurements every 15 s were used to analyze the transient changes in V̇o_2_ at the onset of exercise using the method reported by Rossiter et al. (46) with the following equation:

$$\overset{․}{\text{V}}{\text{O}}_{\text{2}}\left(t\right)=\overset{․}{\text{V}}{\text{O}}_{\text{2}\;\text{BSLN}}+\Delta \overset{․}{\text{V}}{\text{O}}_{\text{2}}\left[1-e^{-\left(t-\text{TD}/\tau \right)}\right]$$

where the onset of exercise is defined as *t* = 0; V̇o_2_ (*t*) represents V̇o_2_ at any time (*t*); V̇o_2 BSLN_ is the baseline V̇o_2_ calculated as an average over 5 min before the onset of exercise; ΔV̇o_2_ is the steady-state increase in V̇o_2_ above the baseline V̇o_2_; *τ* represents the time constant defined as the time required to attain 63% of the ΔV̇o_2_; and TD represents the mathematically determined time delay for V̇o_2_ to start to increase after the onset of exercise. Briefly, after abandoning the phase I response of V̇o_2_ for the first 15 s to exclude the cardiodynamic effect (46), we determined ΔV̇o_2_, *τ*, and TD by fitting the V̇o_2_ data from 15 s to 180 s into the equation so that the sum of the *y* deviation from the equation was minimal (Table 5). We performed the analysis only on the transient V̇o_2_ response from rest to exercise at 0 W, because more than 5-min baseline measurements before increasing workload were reportedly required to determine the parameters (4, 11, 17, 33).

**Table 5.** V̇o_2_ kinetics parameters at the onset of cycling exercise

	CNT		ALA+SFC	
	Before	After	Before	After
Baseline V̇o_2_, ml·kg^−1^·min^−1^	3.0 ± 0.2	3.1 ± 0.1	3.1 ± 0.2	2.7 ± 0.2
ΔV̇o_2_, ml·kg^−1^·min^−1^	4.5 ± 0.3	4.0 ± 0.2	5.1 ± 0.4	3.6 ± 0.2†
*τ*, s	43 ± 4	42 ± 5	43 ± 4	30 ± 4*
TD, s	3 ± 2	6 ± 3	4 ± 2	3 ± 2

Values are means ± SE; *n* = 10 subjects.

ΔV̇o_2_, the steady-state increase in V̇o_2_ above the baseline; *τ*, time constant; TD, time delay.

Compared with before supplement intake:

* *P* < 0.05 and

† *P* < 0.01.

### Training Achievement during the Supplement Intake Period

**Fig. 5.**
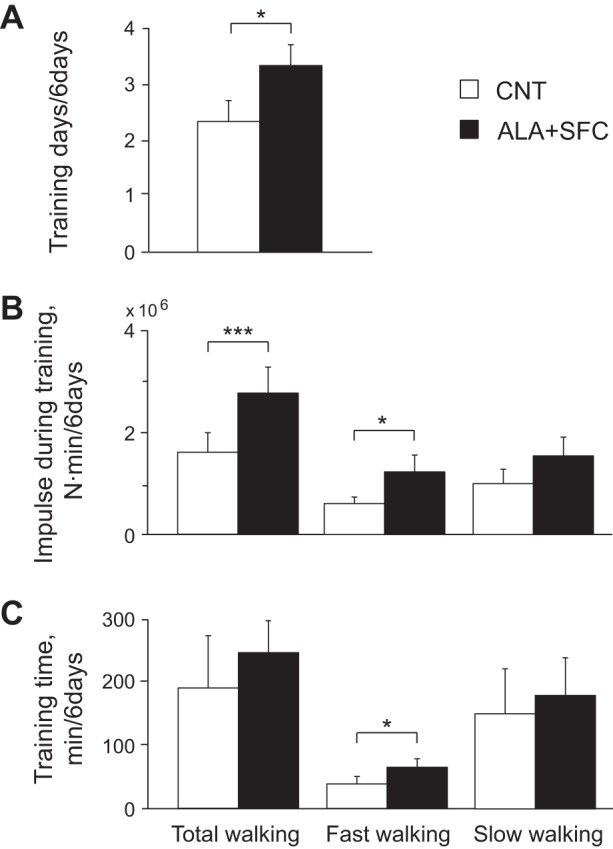
Training days (*A*), training impulse (*B*), and training time (*C*) at total, fast, and slow walking during the supplement intake period. Values are means ± SE of 9 subjects. *P < 0.05 and ***P < 0.001 between the CNT and ALA+SFC trials.

**Fig. 6.**
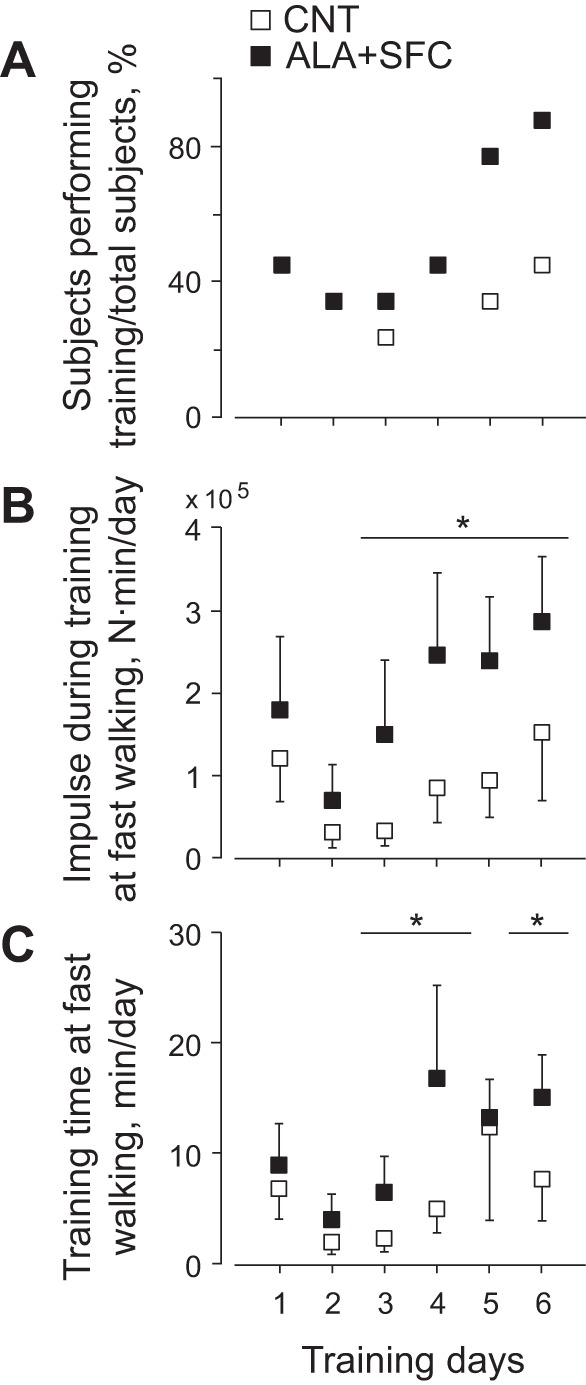
Ratio of subjects performing training to total subjects (*A*), training impulse (*B*), and training time (*C*) at fast walking on each day in the period of supplement intake. Values are means ± SE of 9 subjects. *P <0.05 compared with the CNT trial.

During the supplement intake period, except for the day before the graded cycling test (*days 1–6*), subjects were instructed to perform IWT, repeating five or more sets of 3 min of low-intensity walking at 40% of V̇o_2 peak_, followed by 3 min of high-intensity walking above 70% of V̇o_2 peak_ per day for 4 or more days/wk, where training days, intensity, and time were recorded with a portable triaxial accelerometer (JD Mate) and transferred to the computer server at Shinshu University Graduate School of Medicine over the internet (36). Training intensity was calculated from the product of body weight and the average norm of three-dimensional accelerations per minute and presented as accumulated training impulse (N·min) (24, 52) for 6 days (Fig. 5) and for each day (Fig. 6). Because we failed to record part of the training achievement in 1 of the 10 subjects, training data were presented for the rest of the 9 subjects whose achievements were successfully recorded throughout the intervention.

Regarding the weather conditions on the first 6 of 7 days of supplement intake in the CNT and ALA+SFC trials, the precipitation from 0500 to 2000 was 1.7 ± 0.7 and 2.6 ± 0.8 mm/day, atmospheric temperature was 15.7 ± 1.1 and 13.9 ± 1.0°C, and relative humidity was 71 ± 1 and 75 ± 1%, respectively, with no significant differences observed between trials (all, *P* > 0.1), which suggests no effects of weather conditions on the training achievement.

### Statistics

One-way ANOVA for repeated measures was used to examine any significant differences in physical characteristics and V̇o_2_ kinetics parameters before vs. after the supplement intake period (Tables 1 and 5) and to examine dietary intake in the supplement intake period between trials (Table 3). This model was also used to examine any significant differences in training days, training impulse, and training time during the supplement intake period (Fig. 5). Two-way ANOVA for repeated measures was used to examine any significant differences in variables during the graded cycling test before vs. after the supplement intake period in each trial (Figs. 2–4) and their changes after the period of supplement intake between trials (Table 4). This model was also used to examine any significant differences in training impulse (Fig. 6*B*) and time (Fig. 6*C*) at fast walking and in the weather conditions during the supplement intake period between trials. As a subsequent post hoc test, the Tukey-Kramer test was used to perform any pairwise comparisons between trials. The sign test was used to examine any significant differences in the %ratio of the number of subjects performing walking training to the total number of subjects on each day in the period of supplement intake between trials (Fig. 6*A*), where the data from the CNT and ALA+SFC trials were paired in each subject.

In addition, because this study was conducted in a two-period crossover design, the changes in respiratory responses to graded cycling were further analyzed to test three effects: carryover (physiological and other effects of the first supplement period are still present when the subject enters the second supplement period), period [the effect of stimulation order was present in CNT-(ALA+SFC) sequence group vs. (ALA+SFC)-CNT sequence group], and supplement effects. For the analysis, data were averaged during the graded cycling test, and changes after supplement intake (after-before) were calculated for each period. The three effects were then determined by the method reported by Chow and Liu (8). The null hypothesis was rejected when *P* < 0.05. Values are expressed as means ± SE, unless otherwise indicated.

## RESULTS

As shown in Table 1, body weight, body mass index, HR, and BP at rest remained unchanged after supplement intake in both trials (all, *P* > 0.1). [Hb] before and after supplement intake was 13.3 ± 0.3 and 13.1 ± 0.3 g/dl in the CNT trial, respectively, and 13.1 ± 0.4 and 12.9 ± 0.4 g/dl in the ALA+SFC trial, respectively, with no significant differences between them (all, *P* > 0.1). Additionally, V̇o_2 peak_ and peak HR during the graded cycling test remained unchanged after supplement intake in both trials (both, *P* > 0.3); however, the peak workload tended to increase (*P* = 0.081). Furthermore, the time to exhaustion significantly increased by 0.8 min in the ALA+SFC trial (*P* = 0.022), although not in the CNT trial (*P* = 0.47) (Table 1); the increase in the time to exhaustion was significantly greater in the ALA+SFC than in the CNT trial (*P* = 0.040).

Figure 2 shows the V̇o_2_, V̇co_2_, and V̇e responses during the graded cycling test. When comparing the responses obtained before and after supplement intake, an increase in V̇o_2_ after supplement intake was significantly attenuated by 12% for every workload 1 min after the start of exercise in the ALA+SFC trial (*P* < 0.001), although this did not occur in the CNT trial (*P* = 0.49). Similarly, an increase in V̇co_2_ and V̇e was significantly attenuated by 11% for every workload 2 min after the start of exercise in the ALA+SFC trial (*P* = 0.001 for V̇co_2_, *P* = 0.022 for V̇e), although this did not occur in the CNT trial (*P* = 0.28 for V̇co_2_, *P* = 0.50 for V̇e). We confirmed that the reduction in V̇o_2_ and V̇co_2_ after supplement intake was significantly greater in the ALA+SFC than in the CNT trial (Table 4), but the reduction in V̇e was not different between the trials. The gas exchange threshold before and after supplement intake was 15.2 ± 0.8 and 15.0 ± 0.9 ml·kg^−1^·min^−1^ in the CNT trial, respectively, and 15.9 ± 1.1 and 15.7 ± 0.9 ml·kg^−1^·min^−1^ in the ALA+SFC trial, respectively, with no significant differences between them (all, *P* > 0.4). In addition, there were no significant differences in HR and BP during the graded cycling test before and after supplement intake in either trial (all, *P* > 0.05).

Figure 3 shows [Lac^−^]_p_ during the graded cycling test. After supplement intake, the increase in [Lac^−^]_p_ was significantly attenuated by 16% every workload in the ALA+SFC trial (*P* < 0.001), although this did not occur in the CNT trial (*P* = 0.59). We confirmed that the reduction in [Lac^−^]_p_ after supplement intake was significantly greater in the ALA+SFC than in the CNT trial (Table 4).

The changes in respiratory responses to the graded cycling test were also examined by comparing the CNT-(ALA+SFC) sequence group (*n* = 5) vs. the (ALA+SFC)-CNT sequence group (*n* = 5) (8, 26). For the CNT-(ALA+SFC) sequence, the change in V̇o_2_ after supplement intake was −0.02 ± 0.20 ml·kg^−1^·min^−1^ in *period 1* and −0.96 ± 0.25 ml·kg^−1^·min^−1^ in *period 2*, and the difference over time (*periods 1−2*) was 0.93 ± 0.32 ml·kg^−1^·min^−1^. For the (ALA+SFC)-CNT sequence, the change in V̇o_2_ after supplement intake was −1.06 ± 0.26 ml·kg^−1^·min^−1^ in *period 1* and −0.32 ± 0.45 ml·kg^−1^·min^−1^ in *period 2*, and the difference over time (*periods 1−2*) was −0.74 ± 0.56 ml·kg^−1^·min^−1^, which was significantly different from that observed in the CNT-(ALA+SFC) sequence (*P* = 0.032), thus indicating a significant supplement effect. Similarly, a significant supplement effect was observed in V̇co_2_ (*P* = 0.048) and [Lac^−^]_p_ (*P* = 0.039), but was not observed in V̇e (*P* = 0.13). No carryover or period effects were observed in V̇o_2_, V̇co_2_, V̇e, or [Lac^−^]_p_ (*P* = 0.52–0.97).

Figure 4 shows gross, net, and delta efficiencies during the graded cycling test. When comparing the responses obtained before and after supplement intake, gross and net efficiencies after supplement intake were significantly increased by 12% for every workload in the ALA+SFC trial (*P* = 0.0086 for gross efficiency, *P* = 0.039 for net efficiency), although this did not occur in the CNT trial (*P* = 0.52 for gross efficiency, *P* = 0.34 for net efficiency). On the other hand, we found no significant differences in delta efficiency before vs. after supplement intake in the ALA+SFC trial (*P* = 0.61).

To assess the effect of ALA on transient V̇o_2_ responses to exercise, we determined the V̇o_2_ kinetics parameters at the onset of exercise. As shown in Table 5, we found that *τ* after supplement intake was significantly shortened (*P* = 0.026), and that ΔV̇o_2_ was significantly decreased (*P* = 0.0018) in the ALA+SFC trial, although this did not occur in the CNT trial (both, *P* > 0.2), whereas TD remained unchanged after supplement intake in both trials (both, *P* > 0.1).

Figure 5 shows training days (*A*), training impulse (*B*), and training time (*C*) during the supplement intake period (*days 1–6*). Training days were 42% higher in the ALA+SFC than the CNT trial (*P* = 0.028). The impulses at total and fast walking were 72% and 102% higher in the ALA+SFC than the CNT trial (*P* < 0.001 and *P* = 0.027, respectively). The training time at fast walking was 69% higher in the ALA+SFC than the CNT trial (*P* = 0.039).

Figure 6 shows %ratio of the number of subjects performing training to that of total subjects (*A*), training impulse (*B*), and training time (*C*) on each day in the period of supplement intake. The ratio in the ALA+SFC trial tended to increase after the 5th and 6th days, but with no significant differences from that in the CNT trial (*P* = 0.29 and *P* = 0.22, respectively). Training impulse at fast walking in the ALA+SFC trial increased after the 3rd day, with significant differences from that in the CNT trial (*P* = 0.028). Similarly, training time at fast walking in the ALA+SFC trial increased after the 3rd day, with significant differences from that in the CNT trial (*P* = 0.039).

## DISCUSSION

Although ALA has been reported to enhance the production of heme and cytochromes in vitro (37, 39) to activate complex IV and to increase the ATP production rate in mice (38), there have been no studies conducted to evaluate the effects of ALA on exercise efficiency and home-based walking training achievement in older human subjects. In the present study, we first found in older women that the increases in V̇o_2_, V̇co_2_, and [Lac^−^]_p_ during the graded cycling test were significantly reduced at every workload with ALA+SFC supplementation, and that all of the reductions were significantly greater in the ALA+SFC trial than in the CNT trial. In addition, training days, training impulse, and training time at fast walking were significantly higher in the ALA+SFC trial than in the CNT trial.

### V̇o_2_ during the Graded Cycling Test

As shown in Figs. 2 and 4, the increase in V̇o_2_ during the graded cycling test was significantly reduced by 12% at every workload in the ALA+SFC trial, but not in the CNT trial, with 12% increases in gross and net efficiencies with significance. Regarding the mechanisms, Ferguson et al. (13) suggested that, in *Drosophila melanogaster*, the activity of complex IV, which is one of the enzymes in the mitochondrial ETC, decreased in an older body such that the reduced transferring rate of electrons caused a backlog of electrons in the system, increased the reactive oxygen species generation rate, and decreased the ATP production rate. In fact, biochemical studies in human skeletal muscle suggested that the activity of complex IV decreased from 20 to 80 yr old by 35–50% (45, 50). Additionally, a histochemical study suggested that complex IV-deficient fibers increased with age and were observed in ∼80% of human skeletal muscles at age 80 yr (32). These results suggest that complex IV activity decreases with advanced aging and results in the decreased efficiency of utilizing O_2_ to produce ATP.

Recently, Sacchetti et al. (47) examined the influence of age on cycling efficiency and reported that the gross efficiency was lower in older than young men, regardless of cadence and power output, and it was 13% lower at 60 revolutions/min. Similarly, Bell et al. (5) reported that the net efficiency during cycling was 16% lower at 60 revolutions/min in older than in younger physically active women. Furthermore, Conley et al. (10) examined the factors affecting the reduced cycling efficiency (work/V̇o_2_) with age and suggested that the phosphorylative-coupling efficiency (ATP/V̇o_2_) rather than contractile-coupling efficiency (work/ATP) played a key role in the reduced cycling efficiency with age. In the present study, we first confirmed in the whole body of older human subjects that the oral ingestion of ALA increased the gross and net efficiencies during cycling by 12% at the cadence mentioned above; however, we found no significant increase in delta efficiency, reflecting in part the phosphorylative-coupling efficiency, in the ALA+SFC trial (Fig. 4).

The result of no significant increase in delta efficiency in the ALA+SFC trial might be due to the requirement to determine the value at which V̇o_2_ reached a “steady state” after an increase in workload. If ALA accelerates the O_2_ utilization rate transitionally at an increase in workload, it would not be detected by the steady-state method. We, therefore, analyzed V̇o_2_ kinetics at the onset of exercise and found that *τ* significantly decreased in the ALA+SFC trial, but did not decrease in the CNT trial (Table 5), suggesting that ALA accelerated the O_2_ utilization rate. Additionally, we found that ΔV̇o_2_ significantly decreased in the ALA+SFC trial. Because the product of (*τ* + TD) and ΔV̇o_2_ is an indicator of an O_2_ deficit (4, 46), the decrease is consistent with the attenuated increase in [Lac^−^]_p_ in the ALA+SFC trial, as described below (Fig. 3). These results suggest that ALA accelerates the O_2_ utilization rate to facilitate aerobic ATP production at the onset of exercise.

We analyzed V̇o_2_ kinetics only during a rest-to-work transition using the method reported by Rossiter et al. (46), which is commonly used during a work-to-work transition (17, 33), due to insufficient time for baseline measurements before implementing stepwise increases in workload. However, we found that the difference in the increase in [Lac^−^]_p_ during graded exercise before vs. after supplement intake was enlarged as the workload increased, suggesting that the O_2_ deficit would also decrease at the subsequent stepwise increases of workload in the ALA+SFC trial.

Regarding the relationship between V̇o_2_ kinetics and mitochondrial functions, it has been reported that V̇o_2_ kinetics slow down with aging (17, 33), whereas it is accelerated with improvements in mitochondrial functions after exercise training (4, 11). Additionally, the acceleration of V̇o_2_ kinetics has been suggested to be due to an improved O_2_ utilization rate by the muscle (4, 17, 33). These results suggest that dietary supplementation of ALA improved mitochondrial functions to recover the age-associated decrease in transient O_2_ utilization rates, as well as exercise efficiency determined as work per the total metabolic cost of exercise.

### [Lac^−^]_p_ during the Graded Cycling Test

As mentioned above, an increase in [Lac^−^]_p_ during the graded cycling test was significantly reduced by 16% at every workload in the ALA+SFC trial (Fig. 3), which might be due to the reduced O_2_ deficit. It is well known that [Lac^−^]_p_ is determined by the balance between lactate production and consumption rates, and that [Lac^−^]_p_ starts to increase when the production rate exceeds the consumption rate (7). However, in the present study, it remains unknown whether the reduced [Lac^−^]_p_ during the graded cycling was caused by reduced lactate production rate, enhanced consumption rate, or a combination of the two due to enhanced aerobic capacity.

Another possible mechanism underlying the reduced [Lac^−^]_p_ in the ALA+SFC trial was a reduction in sympathetic nervous activity, the activation of which is known to stimulate glycolysis in active muscles (20) and to limit blood flow to these muscles by suppressing vasodilation due to local metabolites released by the muscles (25). However, this is implausible because we found no significant differences in the HR and BP responses during the graded cycling test between trials. These results suggest that the enhanced aerobic capacity decreased [Lac^−^]_p_ during the graded cycling test, which might also contribute to the prolonged time to exhaustion for the ALA+SFC trial in the present study (Table 1).

### V̇co_2_ and V̇e during the Graded Cycling Test

As demonstrated in Fig. 2, the increase in V̇co_2_ was significantly reduced by a similar degree as V̇o_2_ at every workload during the graded cycling test in the ALA+SFC trial, with a similar V̇e response to V̇co_2_; however, we found no significant differences in the gas exchange threshold before vs. after the ALA+SFC intake period. These results suggest that the attenuated increase in V̇co_2_ for the ALA+SFC trial was caused not by lower respiratory compensation for metabolic acidosis due to the attenuated increase in [Lac^−^]_p_, but by a lower CO_2_ production rate due to a lower o_2_ consumption rate at every workload.

Taken together, these results from laboratory-based experiments suggest that ALA recovers the age-associated decrease in exercise efficiency; however, it remains unknown whether ALA also improves training achievement in the field.

### Training Achievement during the Supplement Intake Period

Until this study, no studies have examined how nutritional supplements affected achievement of home-based walking training. This might be because there is no system that can precisely monitor exercise intensity during home-based walking training, although a higher intensity of aerobic exercise (>50% V̇o_2 peak_) has been recommended in recent guidelines to increase V̇o_2 peak_ in older people (1). To alleviate this difficulty, we recently developed a home-based walking training system that comprises IWT that is programmed according to the individual V̇o_2 peak_ and an information technology network system that tracks the exercise intensity and energy expenditure during training (34, 36, 52). Using this system, in the present study, we found that training days, impulse, and time at fast walking during the supplement intake period significantly increased in the ALA+SFC trial compared with the CNT trial, as summarized in Fig. 5. The precise mechanisms for this remained unclear. However, in the ALA+SFC trial, V̇o_2_ and V̇co_2_ were saved at every workload (Fig. 2), the increase in [Lac^−^]_p_ was significantly attenuated (Fig. 3), and the time to exhaustion was prolonged, and the subjective feeling of fast walking might thus be improved due to reduced panting and muscle pain, ultimately resulting in increases in impulse and time at fast walking in the trial (Fig. 5). This is consistent with a recent study showing that higher muscle mitochondrial efficiency was associated with a faster preferred walking speed in older adults (9).

Moreover, as shown in Fig. 6, the training achievement in the ALA+SFC trial started to increase after the 3rd day of supplement intake, which suggests that at least 3 days are required to attain the effects of ALA+SFC intake in the protocol used in the present study. Although this response seems slow, Mingone et al. (28) reported that, in an organ culture of bovine pulmonary arteries with ALA and iron ion, a significant increase in heme content was observed after 24 h of incubation. They also examined the time-dependent effects of organ culture with ALA on generating protoporphyrin IX, the heme precursor, and reported that at least a 24-h culture period was required to detect the ALA-induced effect. Therefore, in the present study, if the increased training achievement was caused by ALA+SFC-induced increases in heme content and subsequent improvements of mitochondrial function, it seems reasonable to observe these changes after the 3rd day of supplement intake.

Alternatively, the increased training achievement in the ALA+SFC trial might be related to enhanced central mechanisms. ALA administration to rats reportedly induced increased levels of tryptophan and serotonin in the forebrain (12), which might enhance the melatonin concentration in the pineal gland and improve the circadian rhythm in older people (41). Indeed, Rodriguez et al. (44) suggested that, in middle-aged and older subjects, the ingestion of ALA+SFC (50 and 57 mg/day, respectively) for 6 wk improved the quality of sleep, emotional state, and reaction to psychological stress evaluated by questionnaires. Therefore, in the present study, we could not exclude the possibility that the increased training achievement in the ALA+SFC trial was caused by enhanced central mechanisms.

### Limitations

Iron intake per day in the ALA+SFC trial (supplement + dietary intake, Tables 2 and 3) was three times higher than the RDA value (29). Therefore, the possible effects of iron intake on the results were not excluded; however, because subjects showed no symptoms of iron deficiency anemia in the health examination before participating in the study and because [Hb] did not change after ALA+SFC supplementation, it was unlikely that SFC was independently incorporated into the mitochondrial function in the ALA+SFC trial. Therefore, we surmise that the simultaneous ingestion of a mixture of ALA and iron ion is necessary to attain the results, and this interpretation is consistent with several other studies assessing the effects of ALA combined with the iron ion (21, 28, 31, 35, 43).

We employed no sedentary groups in the present study. Because this is the first study to examine whether ALA+SFC supplementation improved exercise efficiency and facilitated training achievement in older people, we started with subjects who were expected to have high and chronic heme demand to compensate for insufficient ATP production during daily IWT. Therefore, it remains unknown whether supplementation would increase the O_2_ utilization efficiency in sedentary subjects.

We used a cycle ergometer to evaluate exercise efficiency, while exercise format in the field experiment was walking. We used this approach because it is more challenging to accurately measure external work done during walking. However, we believe that the improvement in efficiency during cycling observed in the present study is likely to provide some insight into the increased IWT achievement.

In conclusion, the supplementation of ALA+SFC increased exercise efficiency determined as work per the total metabolic cost of exercise and improved voluntary achievement of IWT in older women who had performed habitual training before this study. Because higher training achievement is associated with greater improvements in physical fitness and risk factors for lifestyle-related disease (27), this regimen would be useful to help older women continue habitual exercise training and thus improve their health.

## GRANTS

This study was supported by a grant from the Japan Society for the Promotion of Science (25670117).

## DISCLOSURES

No conflicts of interest, financial or otherwise, are declared by the author(s).

## AUTHOR CONTRIBUTIONS

Author contributions: S.M., A.M., Y.-i.K., S.I., K.T., T.T., M.N., and H.N. conception and design of research; S.M., A.M., Y.-i.K., S.I., Y.K., Y.O., E.S., and H.N. performed experiments; S.M., A.M., and S.I. analyzed data; S.M., A.M., Y.-i.K., S.I., and H.N. interpreted results of experiments; S.M. and A.M. prepared figures; S.M., A.M., and H.N. drafted manuscript; S.M., M.N., and H.N. edited and revised manuscript; S.M., A.M., Y.-i.K., S.I., Y.K., Y.O., E.S., K.T., T.T., M.N., and H.N. approved final version of manuscript.

## References

[1] American College of Sports Medicine. General principles of exercise prescription. In: ACSM's Guidelines for Exercise Testing and Prescription (8th Ed), edited by Thompson WR, Gordon NF, Pescatello LS Baltimore, MD: Lippincott Williams & Wilkins, 2010, p. 152–182.

[2] Åstrand PO, Rodahl K Physical performance. In: Textbook of Work Physiology: Physiological Bases of Exercise (3rd Ed). New York: McGraw-Hill, 1986, chapt. 7, p. 295–353.

[3] Beaver WL, Wasserman K, Whipp BJ A new method for detecting anaerobic threshold by gas exchange. J Appl Physiol 60: 2020–2027, 1986. 3087938 10.1152/jappl.1986.60.6.2020

[4] Bell C, Paterson DH, Kowalchuk JM, Moy AP, Thorp DB, Noble EG, Taylor AW, Cunningham DA Determinants of oxygen uptake kinetics in older humans following single-limb endurance exercise training. Exp Physiol 86: 659–665, 2001. 11571495 10.1113/eph8602209

[5] Bell MP, Ferguson RA Interaction between muscle temperature and contraction velocity affects mechanical efficiency during moderate-intensity cycling exercise in young and older women. J Appl Physiol 107: 763–769, 2009. 19589952 10.1152/japplphysiol.91654.2008 PMC2756006

[6] Brooks GA, Fahey TD, White TP Basics of metabolism. In: Exercise Physiology: Human Bioenergetics and Its Applications (2nd Ed). Mountain View, CA: Mayfield, 1996, p. 37–52.

[7] Brooks GA Lactate: glycolytic end product and oxidative substrate during sustained exercise in mammals: the “lactate shuttle.” In: *Circulation, Respiration, and Metabolism: Current Comparative Approaches*, edited by Gilles R Berlin: Springer-Verlag, 1985, p. 208–218.

[8] Chow SC, Liu JP Statistical inferences for effects from a standard 2 × 2 crossover design. In: Design and Analysis of Bioavailability and Bioequivalence Studies. New York: Dekker, 1992, p. 48–69.

[9] Coen PM, Jubrias SA, Distefano G, Amati F, Mackey DC, Glynn NW, Manini TM, Wohlgemuth SE, Leeuwenburgh C, Cummings SR, Newman AB, Ferrucci L, Toledo FG, Shankland E, Conley KE, Goodpaster BH Skeletal muscle mitochondrial energetics are associated with maximal aerobic capacity and walking speed in older adults. J Gerontol A Biol Sci Med Sci 68: 447–455, 2013. 23051977 10.1093/gerona/gls196 PMC3593613

[10] Conley KE, Jubrias SA, Cress ME, Esselman P Exercise efficiency is reduced by mitochondrial uncoupling in the elderly. Exp Physiol 98: 768–777, 2013. 23085769 10.1113/expphysiol.2012.067314

[11] Daussin FN, Zoll J, Dufour SP, Ponsot E, Lonsdorfer-Wolf E, Doutreleau S, Mettauer B, Piquard F, Geny B, Richard R Effect of interval versus continuous training on cardiorespiratory and mitochondrial functions: Relationship to aerobic performance improvements in sedentary subjects. Am J Physiol Regul Integr Comp Physiol 295: R264–R272, 2008. 18417645 10.1152/ajpregu.00875.2007

[12] Daya S, Nonaka KO, Reiter RJ Melatonin counteracts the 5-aminolevulinic acid-induced rise of rat forebrain tryptophan and serotonin concentrations at night. Neurosci Lett 114: 113–116, 1990. 2381571 10.1016/0304-3940(90)90437-e

[13] Ferguson M, Mockett RJ, Shen Y, Orr WC, Sohal RS Age-associated decline in mitochondrial respiration and electron transport in drosophila melanogaster. Biochem J 390: 501–511, 2005. 15853766 10.1042/BJ20042130 PMC1198930

[14] Frontera WR, Meredith CN, O'Reilly KP, Evans WJ Strength training and determinants of V̇o_2 max_ in older men. J Appl Physiol 68: 329–333, 1990. 2312474 10.1152/jappl.1990.68.1.329

[15] Fueki S, Ueda Y, Dota M, Negishi Y [Development of analytical method of 5-aminolevulinic acid in foods]. Porphyrins 19: 9–14, 2010.

[16] Gaesser GA, Brooks GA Muscular efficiency during steady-rate exercise: effects of speed and work rate. J Appl Physiol 38: 1132–1139, 1975. 1141128 10.1152/jappl.1975.38.6.1132

[17] Grey TM, Spencer MD, Belfry GR, Kowalchuk JM, Paterson DH, Murias JM Effects of age and long-term endurance training on V̇o_2_ kinetics. Med Sci Sports Exerc 47: 289–298, 2015. 24870579 10.1249/MSS.0000000000000398

[18] Handschin C, Spiegelman BM The role of exercise and pgc1alpha in inflammation and chronic disease. Nature 454: 463–469, 2008. 18650917 10.1038/nature07206 PMC2587487

[19] Haskell WL, Phillips WR Effects of exercise training on health and physical functioning in older persons. In: The 1997 Nagano Symposium on Sports Sciences, edited by Nose H, Nadel ER, Morimoto T Carmel, IN: Cooper, 1998, p. 399–417.

[20] Heigenhauser GJF, Parolin ML Role of pyruvate dehydrogenase in lactate production in exercising human skeletal muscle. In: Hypoxia: Into the Next Millennium, edited by Roach RC, Wagner RD, Hackett PH New York: Kluwer Academic/Plenum, 1999, p. 205–218. 10.1007/978-1-4615-4711-2_17 10635003

[21] Higashikawa F, Noda M, Awaya T, Tanaka T, Sugiyama M 5-Aminolevulinic acid, a precursor of heme, reduces both fasting and postprandial glucose levels in mildly hyperglycemic subjects. Nutrition 29: 1030–1036, 2013. 23759263 10.1016/j.nut.2013.02.008

[22] Hoheisel U, Reinohl J, Unger T, Mense S Acidic pH and capsaicin activate mechanosensitive group IV muscle receptors in the rat. Pain 110: 149–157, 2004. 15275762 10.1016/j.pain.2004.03.043

[23] Hood DA Invited review: Contractile activity-induced mitochondrial biogenesis in skeletal muscle. J Appl Physiol 90: 1137–1157, 2001. 11181630 10.1152/jappl.2001.90.3.1137

[24] Iwashita S, Takeno Y, Okazaki K, Itoh J, Kamijo Y, Masuki S, Yanagidaira Y, Nose H Triaxial accelerometry to evaluate walking efficiency in older subjects. Med Sci Sports Exerc 35: 1766–1772, 2003. 14523318 10.1249/01.MSS.0000089350.54959.CB

[25] Kamijo Y, Takeno Y, Sakai A, Inaki M, Okumoto T, Itoh J, Yanagidaira Y, Masuki S, Nose H Plasma lactate concentration and muscle blood flow during dynamic exercise with negative-pressure breathing. J Appl Physiol 89: 2196–2205, 2000. 11090568 10.1152/jappl.2000.89.6.2196

[26] Karst M, Salim K, Burstein S, Conrad I, Hoy L, Schneider U Analgesic effect of the synthetic cannabinoid ct-3 on chronic neuropathic pain: a randomized controlled trial. JAMA 290: 1757–1762, 2003. 14519710 10.1001/jama.290.13.1757

[27] Masuki S, Mori M, Tabara Y, Sakurai A, Hashimoto S, Morikawa M, Miyagawa K, Sumiyoshi E, Miki T, Higuchi K, Nose H The factors affecting adherence to a long-term interval walking training program in middle-aged and older people. J Appl Physiol 118: 595–603, 2015. 25539937 10.1152/japplphysiol.00819.2014

[28] Mingone CJ, Gupte SA, Chow JL, Ahmad M, Abraham NG, Wolin MS Protoporphyrin ix generation from delta-aminolevulinic acid elicits pulmonary artery relaxation and soluble guanylate cyclase activation. Am J Physiol Lung Cell Mol Physiol 291: L337–L344, 2006. 16899710 10.1152/ajplung.00482.2005

[29] Ministry of Health, Labor, and Welfare of Japan. Recommended dietary allowances. In: [Dietary Reference Intakes for Japanese 2010] (1st Ed) Tokyo: Daiichi Shuppan, 2010.

[30] Morikawa M, Okazaki K, Masuki S, Kamijo Y, Yamazaki T, Gen-no H, Nose H Physical fitness and indices of lifestyle-related diseases before and after interval walking training in middle-aged and older males and females. Br J Sports Med 45: 216–224, 2011. 19846423 10.1136/bjsm.2009.064816

[31] Morokuma Y, Yamazaki M, Maeda T, Yoshino I, Ishizuka M, Tanaka T, Ito Y, Tsuboi R Hair growth stimulatory effect by a combination of 5-aminolevulinic acid and iron ion. Int J Dermatol 47: 1298–1303, 2008. 19126021 10.1111/j.1365-4632.2008.03783.x

[32] Muller-Hocker J Cytochrome c oxidase deficient fibres in the limb muscle and diaphragm of man without muscular disease: an age-related alteration. J Neurol Sci 100: 14–21, 1990. 1965203 10.1016/0022-510x(90)90006-9

[33] Murias JM, Kowalchuk JM, Paterson DH Speeding of V̇o_2_ kinetics with endurance training in old and young men is associated with improved matching of local O_2_ delivery to muscle O_2_ utilization. J Appl Physiol 108: 913–922, 2010. 20150562 10.1152/japplphysiol.01355.2009 PMC2853203

[34] Nemoto K, Gen-no H, Masuki S, Okazaki K, Nose H Effects of high-intensity interval walking training on physical fitness and blood pressure in middle-aged and older people. Mayo Clin Proc 82: 803–811, 2007. 17605959 10.4065/82.7.803

[35] Nishio Y, Fujino M, Zhao M, Ishii T, Ishizuka M, Ito H, Takahashi K, Abe F, Nakajima M, Tanaka T, Taketani S, Nagahara Y, Li XK 5-Aminolevulinic acid combined with ferrous iron enhances the expression of heme oxygenase-1. Int Immunopharmacol 19: 300–307, 2014. 24530569 10.1016/j.intimp.2014.02.003

[36] Nose H, Morikawa M, Yamazaki T, Nemoto K, Okazaki K, Masuki S, Kamijo Y, Gen-No H Beyond epidemiology: field studies and the physiology laboratory as the whole world. J Physiol 587: 5569–5575, 2009. 19752116 10.1113/jphysiol.2009.179499 PMC2805369

[37] Ogura S Aminolevulinic acid and porphyrin biosynthesis. In: Aminolevulinic Acid, edited by Okura I, Tanaka T Tokyo: SBI ALApromo, 2011, p. 3–9.

[38] Ogura S, Maruyama K, Hagiya Y, Sugiyama Y, Tsuchiya K, Takahashi K, Abe F, Tabata K, Okura I, Nakajima M, Tanaka T The effect of 5-aminolevulinic acid on cytochrome c oxidase activity in mouse liver. BMC Res Notes 4: 66, 2011. 21414200 10.1186/1756-0500-4-66 PMC3068109

[39] Ota U, Sugihara H, Abe F, Nakajima M, Ogura S, Tanaka T 5-Aminolevulinic acid (5-ALA): a precursor of heme fermentation, metabolism and usage. ALA-Porphyrin Sci 1: 3–17, 2013.

[40] Pedersen BK, Saltin B Evidence for prescribing exercise as therapy in chronic disease. Scand J Med Sci Sports, 16 Suppl 1: 3–63, 2006. 16451303 10.1111/j.1600-0838.2006.00520.x

[41] Reiter RJ Normal patterns of melatonin levels in the pineal gland and body fluids of humans and experimental animals. J Neural Transm Suppl 21: 35–54, 1986. 3018145

[42] Ren JC, Rebrin I, Klichko V, Orr WC, Sohal RS Cytochrome c oxidase loses catalytic activity and structural integrity during the aging process in drosophila melanogaster. Biochem Biophys Res Commun 401: 64–68, 2010. 20833144 10.1016/j.bbrc.2010.09.009 PMC2964050

[43] Rodriguez BL, Curb JD, Davis J, Shintani T, Perez MH, Apau-Ludlum N, Johnson C, Harrigan RC Use of the dietary supplement 5-aminiolevulinic acid (5-ALA) and its relationship with glucose levels and hemoglobin a1c among individuals with prediabetes. Clin Transl Sci 5: 314–320, 2012. 22883608 10.1111/j.1752-8062.2012.00421.x PMC5439781

[44] Rodriguez BL, Shintani T, Harrigan RC Use of the Dietary Supplement 5-Aminiolevulinic Acid (5-ALA) and Its Relationship with Sleep and Mood. NCT01508741 (Online). Clinical Trials.gov, NIH https://clinicaltrials.gov/ct2/show/NCT01508741 [March 2013]. 10.1111/j.1752-8062.2012.00421.x PMC5439781 22883608

[45] Rooyackers OE, Adey DB, Ades PA, Nair KS Effect of age on in vivo rates of mitochondrial protein synthesis in human skeletal muscle. Proc Natl Acad Sci U S A 93: 15364–15369, 1996. 8986817 10.1073/pnas.93.26.15364 PMC26410

[46] Rossiter HB, Ward SA, Doyle VL, Howe FA, Griffiths JR, Whipp BJ Inferences from pulmonary O_2_ uptake with respect to intramuscular [phosphocreatine] kinetics during moderate exercise in humans. J Physiol 518: 921–932, 1999. 10421675 10.1111/j.1469-7793.1999.0921p.x PMC2269465

[47] Sacchetti M, Lenti M, Di Palumbo AS, De Vito G Different effect of cadence on cycling efficiency between young and older cyclists. Med Sci Sports Exerc 42: 2128–2133, 2010. 20386335 10.1249/MSS.0b013e3181e05526

[48] Short KR, Bigelow ML, Kahl J, Singh R, Coenen-Schimke J, Raghavakaimal S, Nair KS Decline in skeletal muscle mitochondrial function with aging in humans. Proc Natl Acad Sci U S A 102: 5618–5623, 2005. 15800038 10.1073/pnas.0501559102 PMC556267

[49] Sohal RS Aging, cytochrome oxidase activity, and hydrogen peroxide release by mitochondria. Free Radic Biol Med 14: 583–588, 1993. 8392019 10.1016/0891-5849(93)90139-l

[50] Trounce I, Byrne E, Marzuki S Decline in skeletal muscle mitochondrial respiratory chain function: possible factor in ageing. Lancet 1: 637–639, 1989. 2564459 10.1016/s0140-6736(89)92143-0

[51] Yamashita N, Watanabe A, Kondo H, Kawata S, Tanaka T, Nakajima M Safety test of a supplement, 5-aminolevulinic acid phosphate with sodium ferrous citrate, in diabetic patients treated with oral hypoglycemic agents. J Funct Foods Health Dis 4: 415–428, 2014.

[52] Yamazaki T, Gen-No H, Kamijo Y, Okazaki K, Masuki S, Nose H A new device to estimate V̇o_2_ during incline walking by accelerometry and barometry. Med Sci Sports Exerc 41: 2213–2219, 2009. 19920753 10.1249/MSS.0b013e3181a9c452

